# Validation of computerized wheeze detection in young infants during the first months of life

**DOI:** 10.1186/1471-2431-14-257

**Published:** 2014-10-09

**Authors:** Lia C Puder, Hendrik S Fischer, Silke Wilitzki, Jakob Usemann, Simon Godfrey, Gerd Schmalisch

**Affiliations:** Department of Neonatology, Charité University Medical Center, Berlin, Germany; Department of Pediatric Pneumology and Immunology, Charité University Medical Center, Berlin, Germany; Emeritus Professor of Pediatrics, Hadassah-Hebrew University, Jerusalem, Israel

**Keywords:** Lung sound, Auscultation, Phonopneumography, Wheezing, Computerized wheeze detection, Infants

## Abstract

**Background:**

Several respiratory diseases are associated with specific respiratory sounds. In contrast to auscultation, computerized lung sound analysis is objective and can be performed continuously over an extended period. Moreover, audio recordings can be stored. Computerized lung sounds have rarely been assessed in neonates during the first year of life. This study was designed to determine and validate optimal cut-off values for computerized wheeze detection, based on the assessment by trained clinicians of stored records of lung sounds, in infants aged <1 year.

**Methods:**

Lung sounds in 120 sleeping infants, of median (interquartile range) postmenstrual age of 51 (44.5–67.5) weeks, were recorded on 144 test occasions by an automatic wheeze detection device (PulmoTrack®). The records were retrospectively evaluated by three trained clinicians blinded to the results. Optimal cut-off values for the automatically determined relative durations of inspiratory and expiratory wheezing were determined by receiver operating curve analysis, and sensitivity and specificity were calculated.

**Results:**

The optimal cut-off values for the automatically detected durations of inspiratory and expiratory wheezing were 2% and 3%, respectively. These cutoffs had a sensitivity and specificity of 85.7% and 80.7%, respectively, for inspiratory wheezing and 84.6% and 82.5%, respectively, for expiratory wheezing. Inter-observer reliability among the experts was moderate, with a Fleiss’ Kappa (95% confidence interval) of 0.59 (0.57-0.62) for inspiratory and 0.54 (0.52 - 0.57) for expiratory wheezing.

**Conclusion:**

Computerized wheeze detection is feasible during the first year of life. This method is more objective and can be more readily standardized than subjective auscultation, providing quantitative and noninvasive information about the extent of wheezing.

## Background

Wheezes consisting of continuous musical sounds of one or more tonal components [1, 2] among the most common adventitious lung sounds in children [3]. Wheezes are usually louder than underlying breath sounds [4] and occur within a broad frequency range [1], with a mean dominant frequency in infants of 225.5 Hz [5]. Wheezing is the acoustic manifestation of lower airway obstruction limiting air-flow in a collapsible tube, thus inducing wall flutter [6]. This phenomenon is usually encountered in asthmatic children [7, 8], but can also occur in children with bronchiolitis [9], cystic fibrosis [10], foreign body aspiration [11], bronchomalacia [12] and primary ciliary dyskinesia [8]. Therefore, detection of wheezing can be useful in diagnosing respiratory disorders and in assessing the efficacy of treatments [9, 13].

Wheezing is most frequently diagnosed by auscultation using a stethoscope or is based on parental reports of wheezes. However, parents often differ in their understanding of wheeze [14, 15] and parentally reported wheezing often cannot be confirmed by auscultation [16]. Moreover, the inter-observer reliability between doctors has been questioned [17, 18] and the quality of auscultation has generally been described as insufficient [4, 7, 19]. This insufficiency is likely due to disparities in the nomenclature used to describe lung sounds [17, 20], in the varying quality of stethoscopes [4, 17] and high noise levels in clinical settings [3]. Computerized lung sound analysis, especially computerized wheeze detection, has been reported to be a more objective and standardizable method, which can overcome the limitations of subjective auscultation [3, 9, 21, 22]. In contrast to auscultation, computerized lung sound analysis can be performed continuously over an extended period of time, and audio recordings can be stored for later assessment and quality monitoring.

To date, few studies have used computerized methods to detect wheezes during the first year of life [9, 23]. The inspiratory and expiratory times are shorter in infants aged <1 year than in older infants, for which cut-off values for the duration of wheezing have been determined [24]. The authors hypothesized that, by determining optimal cut-off values for wheezing, computerized wheeze detection would be an objective, reliable and easy to use method of assessing wheezing also in infants aged <1 year. Therefore the aim of this feasibility study was to determine and validate optimal cut-off values for computerized wheeze detection, based on the assessment by trained clinicians of stored records of lung sounds in infants who recovered after a stay in the neonatal intensive care unit.

## Methods

### Subjects

Computerized wheeze detection and subjective lung sound assessment were performed in 120 infants, of median age 51 postmenstrual weeks, on 144 test occasions. Lung sounds were recorded during lung function testing (LFT) as part of our routine follow-up care of infants requiring intensive care [25]. Patient characteristics are shown in Table 1. Indications for LFT included bronchopulmonary dysplasia (BPD) in 51 infants, respiratory distress syndrome in 35, congenital diaphragmatic hernia in 13, respiratory maladaptation in 6, double aortic arch anomalies in 3, congenital cystic adenomatoid malformation in 2, tracheomalacia in 2 and others in 10.

**Table 1:** **Patient characteristics during the neonatal period and at the time of measurement**, **presented as median** [**interquartile range**] **or n (%)**

*Neonatal period*(*N* = *120*)	
Gestational age (weeks)	30 (26–33)
Birth weight (g)	1483 (775–1930)
Birth weight <1000 g	58 (49%)
Fetal lung maturation^1)^	76/112 (68%)
Male	70 (58%)
Surfactant administration^1)^	21/115 (63%)
*At day of measurement* (*N* = *144*)	
Age (days)	153 (107–273)
Postmenstrual age (weeks)	51 (44.5–67.5)
Body length (cm)	62 (55.0 – 69.125)
Body weight (g)	5995 (4213.75 – 7142.5)

^1)^Numbers reduced due to incomplete data of patients examined by LFT.

All parents provided written informed consent before each LFT, and the study protocol was approved by our Institutional Data Safety Committee.

### Computerized wheeze detection

**Figure 1:**
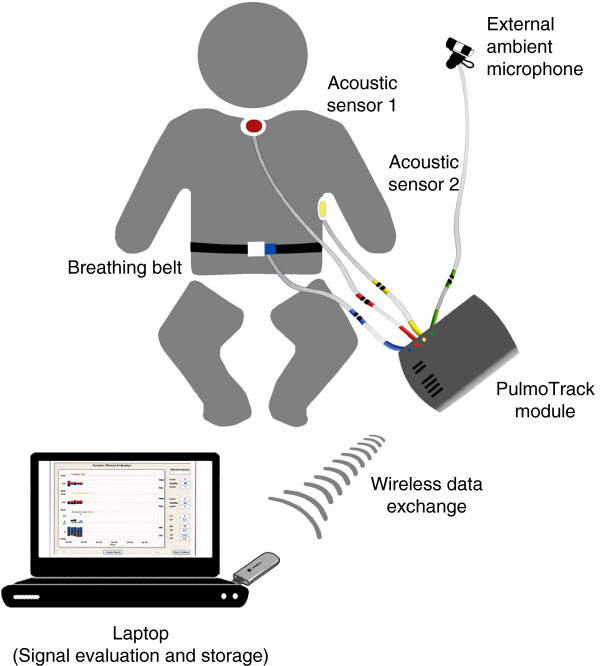
**Measuring principle of the computerized lung sound analysis in neonates.**

Wheezes were detected using the PulmoTrack® Model 2020 (Karmel Sonix Ltd., Israel), an instrument developed for the continuous tracking and recording of breathing sounds and the detection of wheezing. Lung sounds were analyzed using a fast Fourier transform (FFT)-based algorithm for lung sound analysis and two phonopneumographic contact sensors, one applied to the region of the manubrium and the other over the left axillary line (Figure 1). The sensors are coin-shaped piezoelectric elements with linear ±3 dB frequency responses from 75 to 2000 Hz, a resonance at 2.7 kHz and an useable range that extends beyond 4 kHz [9]. The sensors were attached to the skin via adhesive foam pads to reduce ambient noise. Another air-coupled microphone was placed next to each infant to record ambient noises and improve the signal-to-noise ratio, and a respiration belt fitted with tension sensors was strapped around each infant’s chest to detect breathing activity (times of inspiration and expiration). Sound artifacts due to movements of the infant or occasional crying could not totally be eliminated.

The PulmoTrack® calculates the relative inspiratory and expiratory wheeze rates as.


where T_w in/ex_ is the breathing time with wheeze during inspiration/expiration and T_in/ex_ is the total inspiratory/expiratory breathing time.

### Subjective wheeze detection

Recorded sounds coded for each infant were retrospectively evaluated by three medical doctors working in the neonatal intensive care unit and trained before the study using a computer aided instruction on respiratory sounds (R.A.L.E.® Lung Sounds 3.2). Using headphones that minimized surrounding noise, each observer listened to the sound of each infant in a blinded fashion and assessed if wheezing was present or absent, independent of the strength and duration of sounds.

### Measurement protocol

Lung sounds were recorded in clinically stable and sleeping infants who had no respiratory infections during the 3 weeks preceding the tests. Sleep was induced 15–30 min before LFT by oral administration of chloral hydrate (50 mg∙kg^−1^), since sedation was necessary for subsequent more complex LFT [25, 26].

To prevent any interactions lung sound recordings were performed before LFT and before a face mask was applied. Sounds were measured while the infants were supine, with the neck in a neutral position and supported by a neck roll. After attachment of the microphones and breathing belt, an adaptation time of 10–15 min was allowed before lung sounds were recorded. The duration of each recording was 10 minutes. No other lung function tests were performed simultaneously.

### Statistical methods

Patient characteristics and lung sound data are reported as rates (%) or as medians and interquartile ranges (IQR). Incidences of wheezing were compared using Fisher’s exact test. The Kruskal-Wallis rank test was used to investigate the influence of birth weight, mechanical ventilation and BPD on wheeze rates. Inter-observer reliability of lung sound assessment was assessed using Fleiss’ kappa, which is a generalization of Cohen’s kappa to multiple raters that provides a conservative measure of agreement. The 95% confidence interval of Fleiss’ kappa was calculated as described [27], with Fleiss’ kappa scores of 1.0, 0.81–0.99, 0.61–0.80, 0.41–0.60, 0.2–0.40 and <0.2 indicating perfect, almost perfect, substantial, moderate and poor agreement, respectively. Receiver operating characteristic (ROC) curves were calculated to determine the optimal cut-off values for inspiratory and expiratory wheezing times, as measured by the PulmoTrack® and compared with subjective evaluations. All statistical analyses were performed using Statgraphics Centurion® software (Version 16.0, Statpoint Inc., Herndon, VA, USA) and MEDCALC (Version 9.1.0.1, MedCalc Software, Mariakerke Belgium), with p < 0.05 defined as statistically significant.

## Results

### Study population

Lung sounds were recorded in 120 infants on 144 test occasions, with 98 infants (82%) tested on one occasion, 20 (17%) on two occasions and 2 (1.7%) on three occasions. Patient characteristics are shown in Table 1. Most patients (95%) were premature infants with less than 37 gestational weeks and almost half (49%) of all patients were former extremely low birth weight (ELBW) infants, with a birth weight <1000 g. On the day of measurement, their median postmenstrual age (PMA) was 51 weeks, with none requiring any respiratory support.

### Computerized wheeze detection

**Figure 2:**
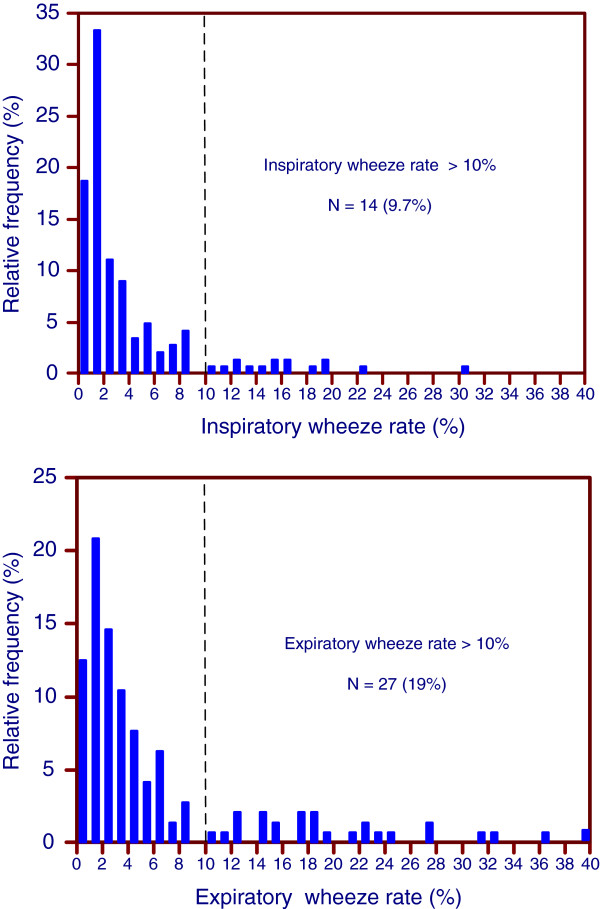
**Distribution of the inspiratory (top) and expiratory (bottom) wheeze rates determined by computerized lung sound analysis using the PulmoTrack®.**

The distributions of inspiratory and expiratory wheeze rates are shown in Figure 2. Both distributions showed a distinct skewness, with maxima at wheeze rates of 1%. The PulmoTrack® detected wheezing in the majority of measurements. Only 27 (19%) of the measurements and 18 (13%) of the expiratory measurements were without wheezing, a difference that was not statistically significant. Wheeze rates >10% were significantly more frequent during expiration than during inspiration (18.8% versus 9.7%, p = 0.042).

### Subjective wheeze detection and inter-observer reliability

Evaluation of wheezing in the 144 recordings by’ the three observers is shown in Table 2. The incidence of expiratory wheezing (36%-50%) was higher than the incidence of inspiratory wheezing (24%-29%). The agreement of the three observers in detection of wheezing was moderate, with Fleiss' kappas (95% confidence interval) of 0.54 (0.52–0.57) for expiratory wheezing and 0.59 (0.57–0.62) for inspiratory wheezing.

**Table 2:** **Lung sounds in investigated infants detected by three observers and the inter-observer variability assessed by Fleiss’ kappa with 95% confidence interval (CI)**

	Observer 1	Observer 2	Observer 3	Fleiss’ kappa(95%CI)
Inspiratory wheezing	35 (24%)	41 (28%)	42 (29%)	0.59 (0.57 - 0.62) (moderate)
Expiratory wheezing	58 (40%)	72 (50%)	52 (36%)	0.54 (0.52 - 0.57) (moderate)

### Cut-off values for computerized wheeze detection

**Figure 3:**
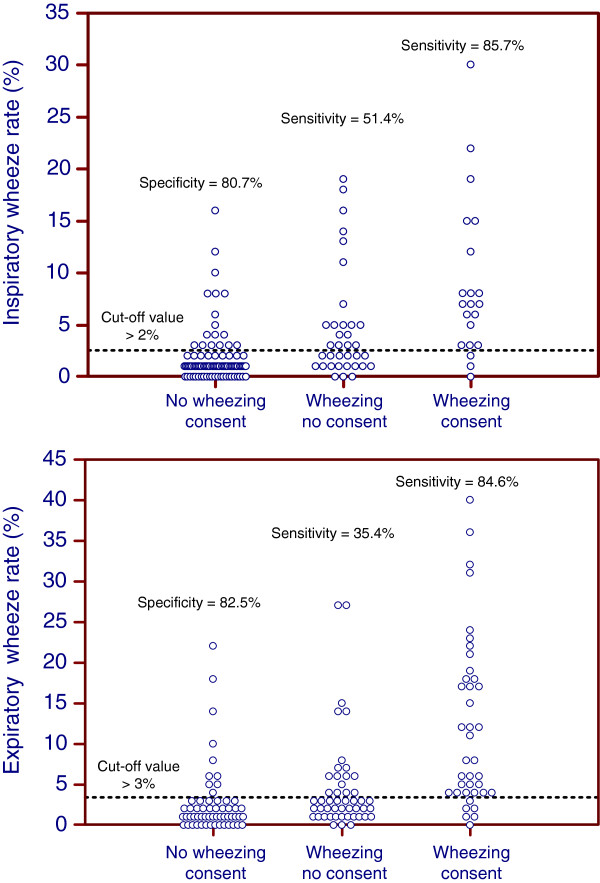
**Specificity and sensitivity of detection of inspiratory (top) and expiratory (bottom) wheezing, based on consensus agreement of three observers of the PulmoTrack® recordings.**

Because the PulmoTrack® detected wheezing in almost all infants, cut-off values for the duration of wheezing were needed to compare computerized and subjective assessments of wheezing. For this purpose, the results of the three observers were classified into three groups: no wheezing detected by all three (group 1), lack of agreement on the presence of wheezing (group 2) and wheezing detected by all three (group 3). Classifications of inspiratory and expiratory wheezing into these three groups are shown in Figure 3. Using ROC analysis, optimal cut-offs for the computer-measured wheezing rates were calculated. The cutoff value for the inspiratory wheezing rate was >2%, which had a sensitivity of 85.7% and a specificity of 80.7%; whereas the cut-off value for the expiratory wheezing rate was >3%, which had a sensitivity of 84.6% and a specificity of 82.5%. For ROC analysis only files in groups 1 and 3 were used. Of the total study population sensitivity and specificity were 85.7% and 71.4% for the inspiratory wheeze detection and 84.6% and 74.3% for the expiratory wheeze detection.

## Discussion

The study showed that the PulmoTrack® can reliably detect wheezing in neonates, with sensitivities of 85.7% for inspiratory and 84.6% for expiratory wheezes and specificities of 80.7% and 82.5%, respectively, using appropriate cut-off values. Computerized wheeze detection reliably detects even short periods of wheezing, as reflected by the low cut-off values of 2% for inspiratory and 3% for expiratory wheezing.

The equipment used in this study differs from that used in previous studies in older children, in which sound was recorded by five sensors [13, 23]. Due to the smaller thoraxes in infants aged <1 year, we used only two chest microphones, as suggested by the developers of the PulmoTrack®. Using five sensor positions simultaneously, the PulmoTrack® has been validated in children aged 6–14 years, with a slightly higher sensitivity (91%) and specificity (89%) in wheeze detection than the consensus by a panel of pulmonary experts who performed auscultation of the same respiratory sounds [13].

The inter-observer reliability for wheeze detection, expressed as the Fleiss Kappa coefficient, was moderate in our study, reflecting a higher inter-observer reliability than reported in most previous studies [17, 28–30]. ROC analysis showed cut-off values of >2% for inspiratory and >3% for expiratory wheeze. In contrast, wheeze rates <5% in older children were not considered clinically significant [24], as healthy children have wheeze rates <5%, with a wheeze rate >10% proposed as a cutoff value [13].

The disparity in lung sound nomenclature has been cited as contributing to disagreements among observers [4, 5, 31]. To prevent this disparity we followed the standardized nomenclature proposed by the American Thoracic Society (ATS) [32] and the International Symposium on Lung Sounds (ILSA) [33]. Although the frequency and duration of wheezes in adults have been defined [32, 34], these definitions are lacking for neonates. Cutoff values in neonates <1 year may differ from those in older children and adults.

This study has several strengths and limitations. The main strengths include the use of a larger sample size than in previous studies on wheeze detection in infants [3, 20] and the use of the same investigators, equipment, and protocol for all patients. Moreover, all the assessed lung sounds were recorded, allowing the three observers to listen to exactly the same sounds. To our knowledge, this study is one of the largest single-center comprehensive studies to compare computerized wheeze detection with the assessment of an expert panel and to analyze inter-observer reliability regarding the detection of wheezing.

One study limitation was that all sound recordings were performed in a quiet lung function testing unit. Thus, we cannot determine the quality of computerized wheeze detection in noisier clinical settings. Moreover, all infants included in our study were sedated for LFT, preventing a determination of the quality of computerized wheeze detection in awake and possibly restless infants.

## Conclusion

Computerized wheeze detection using PulmoTrack® is feasible and reliable in neonates <1 year when using appropriate cut-off values for inspiratory and expiratory wheeze rate. This method provided quantitative and noninvasive information about the extent of wheezing, in contrast to the assessment by trained clinicians, which was subjective and only moderate in the inter-observer agreement. Since this included only infants indicated for LFT due to pulmonary impairment, further studies are needed to evaluate lung sounds in healthy infants.
